# Target competition: transcription factors enter the limelight

**DOI:** 10.1186/gb4174

**Published:** 2014-04-28

**Authors:** Florian A Karreth, Yvonne Tay, Pier Paolo Pandolfi

**Affiliations:** 1 Cancer Research Institute, Beth Israel Deaconess Cancer Center, Department of Medicine and Pathology, Beth Israel Deaconess Medical Center, Harvard Medical School, Boston, MA 02215, USA; 2 Meyer Cancer Center, Weill Cornell Medical College, New York, NY 10021, USA

## Abstract

Transcription factor binding sites compete for a limited pool of bioavailable transcription factor molecules to fine tune gene expression.

## Introduction

The regulation of gene expression is an intriguing and complex topic that has kept scientists busy for decades. Mechanisms that control the transcriptional and translational output of a gene are manifold, but, despite a tremendous amount of progress, the quantitative consequences of gene regulation in response to qualitative changes in the molecular milieu that govern expression are incompletely understood.

Simple artificial genetic circuits have helped to interrogate the intricate interplay between the control and output of gene expression. Genetic circuits have been recreated *in silico* with thermodynamic models, and quantitative measurements have validated that such mathematical models predicted the responses of the circuit under investigation with remarkable accuracy. Thermodynamic models usually operate under the assumption that a transcription factor outnumbers the transcription factor binding sites in the genetic circuit being assessed. While this may often be the case, contexts in which the transcription factor becomes limiting certainly exist.

In a recent issue of *Cell*, Rob Phillips and his team present a thermodynamic model to tackle this problem, which they termed the transcription factor titration effect [1]. Using this model and a simple genetic circuit to validate their predictions, Brewster *et al*. [1] made the intriguing discovery that transcription factor binding sites compete for transcription factors provided that such molecules are not present in excess.

## The transcription factor titration effect

Brewster *et al*. utilized an established regulatory circuit in *Escherichia coli* that consists of the LacI repressor tethered to a mCherry fluorophore and a YFP reporter gene harboring a LacI binding site in its promoter. The authors determined two critical quantities in their thermodynamic model: the number of transcription factor molecules and the number of competing transcription factor binding sites in the experimental regulatory system. A third critical value, the binding affinity of the LacI repressor to specific binding sites, had already been determined in other studies. With these quantities known, the fold-change in YFP expression as a function of the number of LacI repressor molecules could be predicted without any free parameters.

The authors considered several scenarios: one copy of the transcription factor binding site-containing promoter integrated in the bacterial genome, competition between multiple identical promoters in the genome, competition between multiple identical promoters on plasmids with varying copy number, and competition between promoters with varying affinity for the transcription factor on plasmids and a promoter integrated in the genome. These different simulations were validated experimentally and supported the hypothesis that the competition between promoters for transcription factor binding was dictated by the abundance of both the transcription factor and the transcription factor binding site, as well as the affinity of the transcription factor to the promoter.

Interestingly, when the number of transcription factor molecules significantly exceeded the copy number of the promoter, the observed fold-change of reporter gene expression was negligible as all promoters were bound by the transcription factor. Similarly, a much greater number of promoter elements relative to transcription factor molecules had little effect on the fold-change in expression of the reporter gene, because the few available transcription factor molecules were bound by only a small subset of promoters. In other words, if either the transcription factor or its binding site vastly outnumbers the other, competition for binding will not ensue. Indeed, Brewster *et al*. observed that competition was most efficient and the observed fold-change in reporter gene expression was greatest at a state in between the two extreme ends of the spectrum where the number of promoters roughly equals the number of transcription factor binding sites. These findings show for the first time that promoters compete for the binding of transcription factors, provided that optimal molecular conditions are met.

## The many layers of target competition

The study of Phillips and his colleagues is the first to show that transcription factor binding sites compete for the binding of ‘shared’ molecules - transcription factors in their case. This finding describing DNA-protein competition adds a new layer to a phenomenon that we have recently begun to appreciate, namely that various biological entities compete for a limiting pool of binding partners.

Competitive protein-protein interactions are a means to regulate the activity or abundance of individual proteins or protein complexes. For instance, proteins containing an ETGE motif are bound by the adapter protein Keap1 and protect the reactive oxygen species (ROS) detoxifying enzyme Nrf2 from Keap1-mediated destruction by the proteasome [2].

Similarly, protein-RNA competition has been described between various RNA-binding proteins (RBPs) and target RNA transcripts. For example, HuR and AUF1 compete for common target binding sites, thereby regulating mRNA stability [3], [4]. Conversely, mRNAs can sequester specific RBPs from other target transcripts, such as *GAP43* and β-actin, that compete for binding to the ZBP1 RNA binding protein [5].

Finally, we and others have shown competitive RNA-RNA interactions whereby RNA transcripts can compete for a limiting pool of shared microRNAs (miRNAs), a phenomenon that has been demonstrated in plants and mammalian cells, and during virus-host interactions [6]. Interestingly, any RNA molecule containing miRNA-binding sites may operate as so-called competitive endogenous RNAs (ceRNAs), including long non-coding RNAs (lncRNAs), pseudogenes and circular RNAs [6]. This mechanism represents another means to regulate gene expression, and aberrant ceRNA crosstalk has been implicated in the development of cancer [6]. Critically, we observed a similar phenomenon to that described by Brewster *et al*. when we investigated the competition of RNA transcripts for miRNA binding: competition between RNA transcripts was greatest when the various components of the system were present at near-equimolar abundance [7]. These observations suggest that competition involving different molecules - DNA, RNA or protein - follows similar rules.

## Why does it matter?

The transcription factor titration effect has important implications in several contexts. As shown by Brewster *et al*., transcription factor binding sites that are ectopically introduced into cells are able to decoy transcription factors from endogenous genes. Overexpression of complementary DNAs (cDNAs) is one of the most commonly employed techniques to study the function of our favorite genes in cells from unicellular species to mammals. Regardless of whether expression plasmids or viral constructs are used for such experiments, the ectopic introduction of a foreign promoter not only leads to overexpression of the cDNA of interest, it may also result in underexpression of genes from which transcription factors are titrated away. Thus, the results of overexpression experiments need to be interpreted with a grain of salt.

A cellular state where the number of transcription factor binding sites is altered is the cell cycle. Specifically, a cell in the G2 phase of the cell cycle has twice as many promoters as when it was in the G1 phase, and high-affinity transcription factor binding sites should be able to outcompete low-affinity binding sites even more efficiently, especially if the abundance of the transcription factor remains constant during the cell cycle. This may be a mechanism to fine-tune gene expression during mitosis, and warrants further investigations. With the advent of genome editing techniques such as zinc finger nucleases and the CRISPR system, the importance of transcription factor titration in physiology and disease can now be systematically interrogated.

## Target competition in cancer

Another interesting aspect of the work by Brewster *et al*. relates to gene copy number changes that frequently occur in cancers. Genomic amplification or aneuploidy may increase the number of promoters that compete for certain transcription factors by several fold. This, in turn, may drastically alter the transcriptional output of such sequestered transcription factors, especially if the sequestering transcription factor binding site possesses high affinity towards the transcription factor, and promote the development or progression of the disease. One could also imagine that mutations that affect the affinity of transcription factor binding sites alter the transcriptional profile of a cell and, therefore, contribute to cancer development, especially in cases where these binding sites are utilized by multiple transcription factor family members.

**Figure 1:**
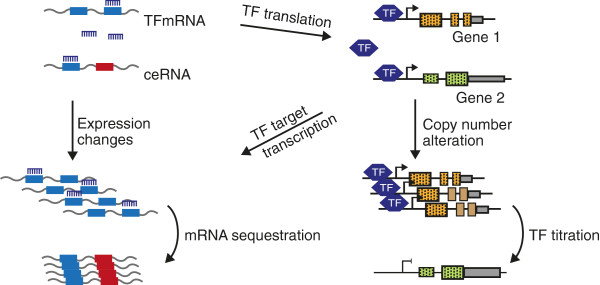
**Target competition: a multi-layered regulatory mechanism.** mRNAs crosstalk through miRNA sequestration resulting in co-expression. In addition, transcription factor mRNAs are translated into transcription factor proteins that can be competed for by transcription factor binding sites in promoters. Moreover, the target genes of such transcription factors are transcribed into RNA transcripts that may also participate in ceRNA crosstalk. TF, transcription factor.

It has been demonstrated that altered ceRNA crosstalk between RNA transcripts may promote cancer development. Such alterations may be due to differential 3′ UTR utilization, translocations, differential gene expression and genomic amplification. Genomic amplification represents an interesting scenario in which the amplified gene on the one hand competes for transcription factors, and on the other hand generates more mRNA molecules that will affect the balance of ceRNA networks. In addition, mRNAs encoding transcription factors can also participate in ceRNA crosstalk (Figure 1). Indeed, it has recently been shown that transcription factor networks and ceRNA networks are intimately intertwined [7].

## Conclusions

It is becoming increasingly clear that target competition affects multiple steps that control the generation of proteins from a DNA blueprint, and that the various molecules involved in target competition form intricate networks that allow for delicate fine-tuning of gene expression. We anticipate a plethora of novel competitive interactions between DNA, RNA and proteins to be discovered and look forward to their functional characterization. The study by Brewster *et al.* has provided an exciting new aspect to the field of target competition and brought us one step closer to a qualitative and quantitative understanding of gene regulation.

## Abbreviations

cDNA: complementary DNA; ceRNA: competitive endogenous RNA; miRNA: microRNA; RBP: RNA-binding protein.

## Competing interests

The authors declare that they have no competing interests.
